# Supplementary material for: Hispidulin Inhibits the Vascular Inflammation Triggered by Porphyromonas gingivalis Lipopolysaccharide
Source: Molecules. 2023 Sep 20;28(18):6717. doi: 10.3390/molecules28186717 (PMC10536826; doi:10.3390/molecules28186717)
Supplement: Supplementary file 1 [file molecules-28-06717-s001.zip › molecules-2611034-supplementary.pdf]

Supplementary Information

The following file contains supplementary material for the paper "Hispidulin inhibits vascular inflammation triggered by *Porphyromonas gingivalis* lipopolysaccharide".

This file is composed of:

- Supplementary figure with supplementary figure legend (1 figures)

Supplementary Figure S1

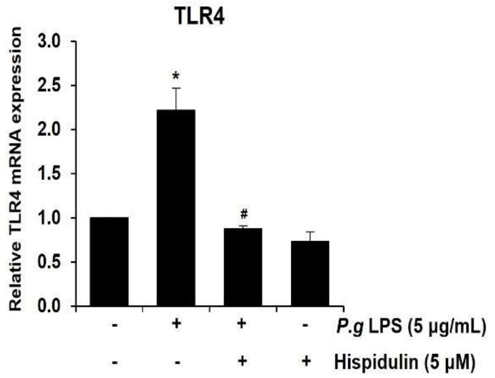

Figure S1. Hispidulin downregulates *P. gingivalis* LPS-induced mRNA expression of TLR4 in endothelial cells. HUVECs were exposed to *P. gingivalis* LPS (5 µg/mL) alone, hispidulin alone (5 µM), or a combination of *P. gingivalis* LPS and hispidulin for 16 h. The expression of TLR4 was analyzed using real-time quantitative PCR. The level in the controls was set to 1.0, and the values were normalized to β-actin. *p <0.01 compared to that in control. #p <0.01 compared to that in *P. gingivalis* LPS.
